# Access and use of digital technology by patients with psychosis at a hospital in South Africa

**DOI:** 10.4102/sajpsychiatry.v29i0.2151

**Published:** 2023-12-07

**Authors:** Smitha Sharma-Misra, Mihoko Maru, Andrew Tomita, Saeeda Paruk

**Affiliations:** 1 Department of Psychiatry, College of Health Sciences, University of KwaZulu-Natal, Durban, South Africa; 2 Department of Psychiatry, Boston Medical Center, Boston, United States; 3 Centre for Rural Health, School of Nursing and Public Health, University of KwaZulu-Natal, Durban, South Africa; 4 KwaZulu-Natal Research Innovation and Sequencing Platform (KRISP), College of Health Sciences, University of KwaZulu-Natal, Durban, South Africa

**Keywords:** information communication technology, digital technology, severe mental illness, schizophrenia, South Africa

## Abstract

**Background:**

There is growing interest in the use of digital information and communication technology (ICT) for mental health care purposes. Information and communication technology tools may enhance mental health literacy and help-seeking behaviour.

**Aim:**

To describe the access to, use and perception of ICT in people with schizophrenia and other psychotic disorders.

**Setting:**

The study was conducted at an urban psychiatric hospital in Durban, KwaZulu-Natal (KZN) province, South Africa.

**Methods:**

Participants completed questionnaires on their socio-demographic characteristics and access to, use and perception of ICT. Multiple ordinal logistic regressions were used to test the association between socio-demographic factors and ICT use and perception.

**Results:**

Of the 165 participants (mean age = 41 years ± 14.2), 54.5% were male, 37.6% were employed, and most (93.3%) lived in an urban area. Most participants (93%) had access to the internet in past 3 months and a smartphone (89.8%). Age (AOR 0.94, *p* = 0.06, CI = 0.88–1.00) and marital status (AOR = 0.26, *p* 0.02, CI = 1.62–253.74) were associated with internet use, while age (AOR = 0.95, *p* 0.03, CI = 0.9–1.00), marital status (AOR = 3.64, *p* = 0.05, CI = 1.03–12.90), income (AOR = 4.02, *p* < 0.01, CI = 1.69–9.54), employment status (AOR = 0.16, *p* < 0.01, CI = 0.06–0.44), and living with HIV (AOR = 5.41, *p* < 0.01, CI = 1.39–21.07) were associated with frequency of internet use. Older participants had lower odds of using a mental health care app (AOR = 0.93, *p* = 0.02, CI= 0.88–0.99). Those with higher incomes had increased odds of seeking mental health information digitally (AOR = 4.33, *p* = 0.03, CI = 1.13–7.54).

**Conclusion:**

People living with psychosis do have access to digital technology although pattern of use maybe influenced by sociodemographic factors.

**Contribution:**

This study provides baseline data on digital technology use in Africa.

## Introduction

The widespread global adoption of information and communication technology (ICT) such as computers, mobile or smart phones, Internet and social media, has significantly transformed the way people communicate over the last several decades. In health care, modern ICT has led to many advances in healthcare technology, including the use of mobile devices for receiving appointment reminders,^1^ monitoring symptoms and health status,^2^ accessing online medical information^3^ and websites or apps that promote illness management.^4,5^ Social media platforms are also frequently accessed by individuals as a means of obtaining medical information or engaging in discussions with other individuals with similar medical conditions or diagnoses.^6^

There is growing interest in the use of ICT in mental healthcare. Studies have explored ICT as a promising method to increase access to evidence-based practices for people with mental health conditions, particularly for those in low-resource settings.^7,8^ Mobile health (mHealth) approaches, for example, have been found to be highly acceptable and feasible among individuals with serious mental illness (SMI).^9^ Additionally, research by Ben-Zeev et al.^10^ suggests their potential to enhance treatment engagement among this population.

Survey studies from high-income countries indicate that ownership of consumer electronics, such as computers or laptops and mobile devices and Internet use among people with SMI is comparable to the general population.^11,12,13,14,15,16^ Research to date also shows that people with SMI frequently use the Internet to seek mental health information (e.g. medication) and resources, like support groups and other community-based mental health services and programmes.^11,15,17,18^ Internet users with SMI are also active users of social media, such as YouTube and Facebook to connect with others.^11,16^

Existing research has also investigated the correlates of Internet access and use among people with SMI. People who are younger and with higher level of education are more likely to use the Internet, while lack of telecommunication infrastructure, financial resources to afford the cost of Internet or the skills or knowledge to use the Internet, have been identified as barriers to Internet use.^14,15,16^ People with SMI who have a comorbid substance use disorder also are less likely to use the Internet compared to those without a substance use disorder.^19^

Data on ICT access and use among individuals living with SMI is limited in low- and middle-income countries (LMICs), including South Africa. Exploring the use of among individuals with SMI in South Africa may help lay the groundwork for developing ICT-based tools to improve mental health literacy, support help-seeking behaviour and enhance mental healthcare access in this population. This study, therefore, aimed to understand access, use and perception of ICT among people with schizophrenia spectrum and other psychotic disorders in KwaZulu-Natal (KZN) province, South Africa.

## Research methods and design

### Study design, setting, participants and procedure

This study used a descriptive, cross-sectional survey design. Adult patients with schizophrenia spectrum and other psychotic disorders who were receiving treatment at a psychiatric hospital in Durban, KZN province, South Africa, were recruited and asked to complete a one-time survey. The psychiatric hospital serves diverse multicultural communities in eThekwini and surrounding municipalities, and provides both inpatient and outpatient services. Patients who were: (1) 18 years and older with a diagnosis of schizophrenia spectrum and other psychotic disorders, as per the *Diagnostic and Statistical Manual of Mental Disorders Fifth Edition* (DSM-5) criteria,^20^ (2) receiving care at the psychiatry outpatient department, (3) in treatment for at least a 6-months and (4) able to speak or write in English, were invited to participate. All patients who were acutely psychotic or had a comorbid intellectual disability were excluded from the study. The questionnaires were completed by the participants while they were alone in a private room at the hospital to maintain social distancing because of coronavirus disease 2019 (COVID-19) restrictions. All questionnaire items were written in English. Data were collected between August 2021 and November 2021.

### Measures

The survey measures consisted of a socio-demographic and clinical questionnaire and an instrument to assess participants’ access, use and perception of technology and social media. The demographic data included participants’ age, gender, marital status, race, educational levels, occupational history and household income. Comorbid medical, psychiatric illnesses and substance use history were also collected, which were later confirmed through a review of their clinical records.

Participants’ patterns of technology access and use and their perception towards technology used for health-related purposes were measured using the New Technologies and Mental Health Survey (NTMHS).^21^ The measure is a structured questionnaire that assess the following: (1) Internet access and frequency of use, (2) electronic device ownership, (3) use of social media, (4) use of Internet for seeking information on mental health-related content, (5) participants’ experience and beliefs about using mobile technology and Internet for mental health reasons and (6) participants’ interest toward using e-health technology for their mental health care. The questionnaire does not include any subscales or a total composite score; only responses to individual items are reported. At the time this study was conducted, there were no culturally and linguistically adapted measures validated for the South African population to measure the construct of technology use for adults with SMI. Permission to use the tool was obtained from the corresponding author.

### Data analysis

Two analyses were undertaken for this study, firstly, summarising their socio-demographic and clinical characteristics, as well as their access and use of technology using descriptive statistics. Secondly, we fitted regression models to identify socio-demographic and clinical correlates of: (1) Internet use, (2) frequency of Internet use, (3) smart phone ownership, (4) willingness to use a mental health app on their smart phone and (5) history of seeking mental health information on the internet. Logistic regressions were fitted in all the above-mentioned dependent variables with binary responses (with the exception of frequency of Internet use, where ordinal logistic regression was fitted). The regression models controlled for socio-demographic and clinical correlates consisting of age, sex, marital status, educational attainment, household income, employment status, residential area (i.e. urban vs. rural), length of living with mental health (in years) and lifetime alcohol or cannabis use. The study data were analysed using Stata v17.0 (Stata Corporation, Stata Statiscal Software release 17, Colledge station, Texas Stat corporation LP, 2017).

### Ethical considerations

The study was approved by the Biomedical Research Ethics Committee of the University of KwaZulu-Natal (reference number BE013/18), the KZN Department of Health, and the hospital manager. Written informed consent was obtained from all participants prior to data collection.

## Results

### Socio-demographic profile

Table 1 summarises the socio-demographic characteristics of the participants. A total of 165 participants were enrolled in this study. Participants’ mean age was 41 years (standard deviation [SD] = 14.2). There were slightly more male (54.5%) than female participants (45.5%), with more than half being unmarried (54.5%). Most did not have any tertiary education, and about two-thirds were either unemployed or on disability grants. The majority (93.3%) lived in urban areas, 12.0% reported that they were living with HIV and about half consumed alcohol and 40.0% used cannabis.

**TABLE 1:** Socio-demographic characteristics of the study participants (*n* = 165).

Variable	Mean	s.d.	*N*	%
**Age (years)**	41	14.2	-	-
**Length of psychiatric illness (years)** †	10	-	-	-
**Sex**	-	-		
Feale	-	-	75	45.5
Male	-	-	90	54.5
**Marital Status**				
Single	-	-	90	54.5
Married	-	-	53	32.1
Divorced, separated or widowed	-	-	22	13.3
**Tertiary education**				
No	-	-	127	77.0
Yes	-	-	38	23.0
**Household income per month**				
R0.00–R4999.00	-	-	52	31.5
R5000.00–R9999.00	-	-	55	33.3
R10 000 or more	-	-	58	35.2
**Occupational history**				
Employed or self-employed	-	-	62	37.6
Unemployed	-	-	48	29.1
On disability grant	-	-	55	33.3
**Residential area**				
Urban	-	-	154	93.3
Rural	-	-	11	6.7
**Living with HIV**				
No	-	-	145	87.9
Yes	-	-	20	12.1
**Alcohol use (past 3 months)**				
No	-	-	79	47.9
Yes	-	-	86	52.1
**Cannabis (past 3 months)**				
No	-		102	61.8
Yes	-	-	63	38.2

IQR, interquartile range; HIV, human immunodeficiency virus; s.d., standard deviation.

† , IQR = 5–17

### Access and use of information and communication technology

Table 2 summarises mobile phone ownership and patterns of digital technology use among the participants. The majority of the participants (83%) used the Internet in the last 3 months with close to half of the participants (48.4%) reporting daily use. Most participants (93.9%) owned some type of electronic device (e.g. mobile, computer) to access the Internet. Almost two thirds reported that they have used the Internet to seek information about mental health, including symptoms (18.2%) and medication side effects (0.6%). Most indicated that the information gathered from the Internet did not influence them to discontinue medication. Communication (e.g. WhatsApp) and social media use (e.g. Facebook) via mobile technology were also common among the participants (88.5% and 83.0%, respectively). Responses to items on their experience and perception towards ICT use for mental health reasons revealed that most participants (71.5%) agreed that Internet use was beneficial to their mental health and expressed a high interest in owning a mobile phone application that would help cope with mental illness (81.4%).

**TABLE 2:** Access and use of technology by study participants.

Variable	*N*	%
**Accessed the Internet in the last 3 months:**		
No	28	17.0
Yes	137	83.0
**Frequency of access to Internet:**		
Monthly, less than once a week	31	19.3
Weekly, but not everyday	52	32.3
Daily, at least five times a week	78	48.4
**Own an electronic device (mobile, computer, laptop, tablet, etc.) to access Internet:**		
No	10	6.1
Yes	155	93.9
**Own or share personal electronic device:**		
I own a personal device	138	91.4
I share a device with a relative	13	8.6
**Mobile phone type:**		
Traditional mobile phone	16	10.2
Smartphone (with access to Internet)	141	89.8
**Mobile phone services used in the last 3 months to send messages (WhatsApp, SMS, etc.):**		
No	19	11.5
Yes	146	88.5
**Used a social media platform (Facebook, Instagram, Twitter, etc.):**		
No	28	17.0
Yes	137	83.0
**I have sought information about mental health on the Internet.**		
No	58	35.2
Yes	107	64.8
**I have sought information on mental health symptoms on the Internet.**		
No	135	81.8
Yes	30	18.2
**I have sought information on medication side effects for mental health on the Internet.**		
No	164	99.4
Yes	1	0.6
**I stopped taking medication because of information I read on the Internet.**		
Strongly agree	0	0
Somewhat agree	0	0
Neutral	3	1.8
Somewhat disagree	90	54.5
Strongly disagree	72	43.6
**As a whole, I think the Internet is beneficial to my mental health.**		
Strongly agree	31	18.8
Somewhat agree	87	52.7
Neutral	16	9.7
Somewhat disagree	23	13.9
Strongly disagree	8	4.8
**I am interested in owning a smartphone app to help cope with mental illness.**		
No	29	18.6
Yes	127	81.4

Table 3 describes the association of socio-demographic and clinical factors with Internet use and frequency of use, smart phone ownership, willingness to use a mental health e-health app and history of seeking mental health information on the Internet. Compared to those who were divorced, separated or widowed, those who were married had 21 times the odds (AOR = 20.76, *p* = 0.02) of using the Internet in the last 3 months. Those with income between R5000.00 and R9999.00 (vs. <R5000.00) also had higher odds of using the Internet (AOR = 7.05, *p* = 0.02), while those living in rural (vs. urban) settings had 93% lower odds of recent internet use (AOR = 0.07, *p* = 0.02).

**TABLE 3:** Regression model on Internet use, frequency, mobile phone ownership and attitudes towards information and communication technology use.

Variable	Internet use in the last 3 month					Frequency of internet use					Posession of Smart Phone					Willingness to use mental health app on smart phone					History of seeking mental health info on internet				
	OR	aOR	95% CI		*P*	OR	aOR	95% CI		*P*	OR	aOR	95% CI		*P*	OR	aOR	95% CI		*P*	OR	aOR	95% CI		*P*
**Sex**																									
Female	(Ref)	(Ref)	(Ref)	(Ref)	(Ref)	(Ref)	(Ref)	(Ref)	(Ref)	(Ref)	(Ref)	(Ref)	(Ref)	(Ref)	(Ref)	(Ref)	(Ref)	(Ref)	(Ref)	(Ref)	(Ref)	(Ref)	(Ref)	(Ref)	(Ref)
Male	1.48	1.45	0.32	6.56	0.63	0.80	1.88	0.76	4.66	0.17	1.64	2.12	0.45	9.97	0.34	1.36	1.10	0.32	3.81	0.87	0.78	1.08	0.37	3.11	0.89
**Age (years)**	0.93	0.94	0.88	1.00	0.06	**0.96**	**0.95**	0.90	1.00	**0.03**	0.95	0.94	0.87	1.01	0.10	**0.94**	**0.93**	0.88	0.99	**0.02**	0.98	1.02	0.96	1.07	0.53
**Marital Status**																									
Divorced, separated, widowed	(Ref)	(Ref)	(Ref)	(Ref)	(Ref)	(Ref)	(Ref)	(Ref)	(Ref)	(Ref)	(Ref)	(Ref)	(Ref)	(Ref)	(Ref)	(Ref)	(Ref)	(Ref)	(Ref)	(Ref)	(Ref)	(Ref)	(Ref)	(Ref)	(Ref)
Single	2.97	0.38	0.06	2.54	0.32	2.72	0.94	0.24	3.65	0.93	2.93	1.02	0.14	7.33	0.99	2.75	0.32	0.07	1.58	0.16	1.37	0.77	0.18	3.32	0.72
Married	**17.65**	**20.26**	1.62	253.74	**0.02**	**7.00**	**3.64**	1.03	12.90	**0.05**	5.21	5.60	0.77	40.50	0.09	4.82	1.76	0.37	8.30	0.48	4.89	1.93	0.45	8.28	0.37
**Tertiary Education**																									
No	(Ref)	(Ref)	(Ref)	(Ref)	(Ref)	(Ref)	(Ref)	(Ref)	(Ref)	(Ref)	(Ref)	(Ref)	(Ref)	(Ref)	(Ref)	(Ref)	(Ref)	(Ref)	(Ref)	(Ref)	(Ref)	(Ref)	(Ref)	(Ref)	(Ref)
Yes	9.99	3.22	0.24	42.90	0.38	9.07	3.30	0.99	11.02	0.05	0.67	0.36	0.06	2.29	0.28	1.29	0.41	0.09	1.91	0.26	14.20	3.92	0.71	21.55	0.12
**Household Income**																									
< R5000.00	(Ref)	(Ref)	(Ref)	(Ref)	(Ref)	(Ref)	(Ref)	(Ref)	(Ref)	(Ref)	(Ref)	(Ref)	(Ref)	(Ref)	(Ref)	(Ref)	(Ref)	(Ref)	(Ref)	(Ref)	(Ref)	(Ref)	(Ref)	(Ref)	(Ref)
R5000.00-R9999.00	**5.76**	**7.05**	1.39	35.73	**0.02**	**5.66**	**4.02**	1.69	9.54	**<0.01**	2.92	1.95	0.38	10.04	0.43	3.08	2.72	0.79	9.39	0.11	**4.23**	**2.91**	1.13	7.54	**0.03**
R10 000.00 and higher	7.77	2.02	0.38	10.69	0.41	**12.52**	**4.50**	1.58	12.84	**<0.01**	1.49	0.95	0.16	5.61	0.95	3.28	3.38	0.74	15.43	0.12	**9.46**	**4.33**	1.34	14.02	**0.01**
**Employment Status**																									
Employed	(Ref)	(Ref)	(Ref)	(Ref)	(Ref)	(Ref)	(Ref)	(Ref)	(Ref)	(Ref)	(Ref)	(Ref)	(Ref)	(Ref)	(Ref)	(Ref)	(Ref)	(Ref)	(Ref)	(Ref)	(Ref)	(Ref)	(Ref)	(Ref)	(Ref)
Unemployed	0.07	0.27	0.02	3.34	0.30	0.17	0.38	0.14	1.01	0.05	0.92	1.79	0.27	11.63	0.54	0.40	0.88	0.22	3.54	0.86	**0.07**	**0.13**	0.03	0.48	**<0.01**
On disability grant	0.03	0.16	0.01	1.82	0.14	**0.06**	**0.16**	0.06	0.44	**<0.01**	0.53	0.84	0.14	5.11	0.85	0.43	1.27	0.30	5.46	0.75	**0.05**	**0.14**	0.04	0.5	**<0.01**
**Residential area**																									
Urban	(Ref)	(Ref)	(Ref)	(Ref)	(Ref)	(Ref)	(Ref)	(Ref)	(Ref)	(Ref)	(Ref)	(Ref)	(Ref)	(Ref)	(Ref)	(Ref)	(Ref)	(Ref)	(Ref)	(Ref)	(Ref)	(Ref)	(Ref)	(Ref)	(Ref)
Rural	**0.52**	**0.07**	0.01	0.68	**0.02**	0.48	0.39	0.08	1.91	0.25	0.48	0.13	0.01	1.32	0.09	1.03	0.40	0.05	3.43	0.40	0.42	0.33	0.06	1.95	0.22
**Length of living with me ntal illness (Number of years)**	0.89	0.95	0.85	1.06	0.34	0.93	1.01	0.94	1.09	0.76	0.94	1.00	0.90	1.11	0.94	0.92	0.98	0.90	1.06	0.60	0.95	0.96	0.88	1.04	0.31
**Living with HIV**																									
No	(Ref)	(Ref)	(Ref)	(Ref)	(Ref)	(Ref)	(Ref)	(Ref)	(Ref)	(Ref)	(Ref)	(Ref)	(Ref)	(Ref)	(Ref)	(Ref)	(Ref)	(Ref)	(Ref)	(Ref)	(Ref)	(Ref)	(Ref)	(Ref)	(Ref)
Yes	4.35	14.11	0.79	253.70	0.07	**1.50**	**5.41**	1.39	21.07	**0.01**	2.34	7.03	0.39	125.89	0.19	4.93	7.17	0.63	81.02	0.11	1.30	2.28	0.50	10.42	0.29
**Lifetime alcohol use**																									
No	(Ref)	(Ref)	(Ref)	(Ref)	(Ref)	(Ref)	(Ref)	(Ref)	(Ref)	(Ref)	(Ref)	(Ref)	(Ref)	(Ref)	(Ref)	(Ref)	(Ref)	(Ref)	(Ref)	(Ref)	(Ref)	(Ref)	(Ref)	(Ref)	(Ref)
Yes	2.71	1.78	0.37	8.45	0.47	1.18	1.57	0.64	3.87	0.33	2.06	1.14	0.26	5.04	0.87	1.80	1.08	0.33	3.50	0.90	1.14	2.12	0.71	6.28	0.18
**Lifetime cannabis use**																									
No	(Ref)	(Ref)	(Ref)	(Ref)	(Ref)	(Ref)	(Ref)	(Ref)	(Ref)	(Ref)	(Ref)	(Ref)	(Ref)	(Ref)	(Ref)	(Ref)	(Ref)	(Ref)	(Ref)	(Ref)	(Ref)	(Ref)	(Ref)	(Ref)	(Ref)
Yes	2.07	1.92	0.32	11.50	0.48	0.76	0.56	0.20	1.54	0.26	1.45	0.67	0.11	3.96	0.66	1.82	1.48	0.37	5.92	0.58	0.73	0.77	0.23	2.62	0.68

Ref, Reference bold; HIV, human immunodeficiency virus.

Note: Values in bold font indicate significant findings at either *p* < 0.05 or *p* < 0.01 for the OR.

Regarding the frequency of Internet use, participants’ age, marital status, income, employment and HIV status were significantly associated. A 1 year increase in age was associated with 5% lower odds of more frequent Internet use (AOR = 0.95, *p* < 0.01). However, compared to being in an income group of <R5000.00, being in higher income groups (AOR = 4.02, *p* < 0.01 for R5000.00–R9999.00; AOR = 4.50 for R10 000.00 or higher, *p* < 0.01) was associated with higher odds of more frequent Internet use. Those who were married (vs. divorced, separated or widowed) and those living with HIV (vs. not living with HIV) were also associated with 3.6 times the odds (AOR = 3.64, *p* = 0.01) and 5 times the odds (AOR = 5.41, *p* = 0.05) of more frequent Internet use, respectively. On the other hand, those on a disability grant had lower odds of frequent Internet use (AOR = 0.16, *p* < 0.01).

Age was also associated with people’s willingness to use a mental health e-health app on a smart phone; each year increase in age was associated with 7% lower odds of using an app (AOR = 0.93, *p* = 0.02). Furthermore, those with higher incomes had higher odds (AOR = 2.91 for R5000.00–R9999.00; AOR = 4.33 for R10 000.00 or higher, *p* < 0.01) of having a history of seeking mental health information on the Internet than those in the income group of <R5000.00. On the contrary, those who were unemployed or on a disability grant (vs. employed participants) had lower odds (AOR = 0.13, *p* < 0.01 for unemployed; AOR = 0.14, *p* < 0.01 for disability grant recipients) of seeking mental health information on the Internet. There were no significant demographic predictors of smart phone ownership.

## Discussion

A cross-sectional survey study was conducted with 165 patients with schizophrenia spectrum and other psychotic disorders in KZN province of South Africa. Results showed that most people in this sample of participants owned or had access to an ICT device, had access to the Internet and used the Internet at least once a week. Internet was commonly used to seek information on mental health online and to access communication apps or social media platforms. Overall, participants had a positive perception toward using Internet as a means to support their mental health; these findings are consistent with descriptive studies on Internet use among people with SMI from high-income countries.^11,15,16,22^

Results from the logistic regressions were also comparable to existing research, with the odds of using the Internet being associated with one’s marital status and income level. People who are married may be living with a spouse or have a partner who has a mobile device and thus have additional options or resources to access the Internet and may also explain the increased odds found in the frequency of Internet use. The finding that living in a rural residential area decreases the odds of Internet use is likely because of lack of telecommunication infrastructure. The finding relating to increased odds of using the Internet in middle compared to higher income groups is not clear but may be confounded by other factors, such as age and education level.

Our findings did not show that older age was associated with lower odds of Internet use like in other studies,^15^ but was significantly associated with lower odds of Internet use *frequency*. One possible explanation is that pattern and reason for use may differ. This will need to be further explored in a mixed method study.

Living with HIV and SMI also increased the odds of frequent Internet use, which may be attributable to increased information seeking behaviours associated with this subgroup of people who have a chronic medical comorbid disorder.

Brunette et al.^23^ reported in their study that younger age predicted willingness to use a mobile programme for mental health among adults (age 18+) with SMI. The study also found that a 1-year increase in age was associated with lower odds of being willing to use a mental health app on a smartphone. Income and employment status were also associated with participants’ history of seeking mental health information online. Having a higher income increased the odds, while being unemployed or being on a disability grant lowered the odds of ever having sought mental health information online. Given that those who have higher income had higher odds of using the Internet, it is not surprising that participants sought information online to learn about mental health. Similarly, being on a disability grant decreased the odds of frequent Internet use, which may also reflect the lower odds of using the Internet to search for mental health information among this subgroup. If the opportunities to use the Internet are less frequent, these people may be using the Internet for content that is more relevant to their needs or interests.

The study findings provide empirical support for expanding the use of digital technology in mental health care or ‘E-mental health’ in LMICs settings. It is evident, however, that age is a significant factor that has an impact on the uptake of mobile technology. Furthermore, disparities exist where those with low-income or living in rural areas lack access to Internet and other ICT. Integrating digital interventions in mental health care is complex, and despite emerging evidence for effective mental health apps, user retention rates for health apps are low among the general population and are even lower for people with SMI.^24^ There are also ethical concerns that people with SMI who may have limited digital knowledge, technical skills or cognitive abilities are at increased risk for online harm.^25^ More studies are therefore needed in the context of LMICs to understand the feasibility and safety of integrating digital technology into mental health services.

### Limitations

The study has a number of limitations. Because this study was conducted in a large urban hospital, the findings may not be generalisable to other settings or populations with SMI, such as rural areas or other types of medical health care facilities or to non-English speaking patients. The study was only able to include those who can read or write English as the validated measure were only available in English at the time of this study. The NTMHS was also not culturally adapted to be used in the South African context that included questions that were relevant to the national or local culture and environmental factors that influence ICT use and access. Furthermore, a larger sample size may have yielded more precise associations in the logistic regression models. There may also have been under or over-reporting on the NTMS, as the study relied on self-report by participants. Future research using a measure validated for the South African population, and a mixed methods approach with longitudinal data as well as qualitative data would strengthen the results and help understand the mechanisms underlying the associations identified in the present study.

## Conclusion

The ICT access, use and perceptions of adults with SMI in LMICs are an area of mental health research that has received inadequate attention but has implications on clinical practice. This study suggests people living with psychosis do have access and use digital technology. Hence, this may pave the way for exploring the advancement of digital mental health technologies, in low-resource settings where community-based programmes and access to mental health information is limited.
